# Fifty years of primary health care in the rainforest: temporal trends in morbidity and mortality in indigenous Amerindian populations of Suriname

**DOI:** 10.7189/jogh.08.020403

**Published:** 2018-12

**Authors:** Marthelise GM Eersel, Stephen GS Vreden, Edward D van Eer, Dennis RA Mans

**Affiliations:** 1 Department of Public Health, Faculty of Medical Sciences, Anton de Kom University of Suriname, Paramaribo, Suriname; 2 Department of Internal Medicine and Infectious Diseases, Academic Hospital Paramaribo, Paramaribo, Suriname; 3 Medische Zending Primary Health Care Suriname, Paramaribo, Suriname; 4 Department of Pharmacology, Faculty of Medical Sciences, Anton de Kom University of Suriname, Paramaribo, Suriname

## Abstract

**Background:**

The Amazonian Amerindian populations living in the southern and southwestern hinterlands of Suriname (South America) have come into contact with western health care since approximately fifty years ago. In this study, secondary data were used to assess the impact of Medical Mission’s fifty-year old primary health care program on the health status of these populations.

**Methods:**

Using data from the primary health care facilities of Medical Mission for 1965-1970, 1973-1977, 1982-1985, and 1997-2014, temporal trends in incidence and mortality of respiratory tract infections, gastroenteritis, and malaria; population composition; birth and death rates; and polyclinic consultations in these communities have been assessed over the period between 1965 and 2014.

**Results:**

In the period covered by this study, the incidence of respiratory tract infections and gastroenteritis declined by about 75% and 53%, respectively, while malaria incidence rose sharply from the 1980s through 2005 but subsequently declined to levels approximating elimination. Crude death rates dropped by about 70% while birth rates declined by about 50% in the 1980s and since then remained at this level. The population doubled in size and increased in all age groups, particularly in the age group of ≥59 years. The infant mortality rate declined by 50%. In addition, the average yearly number of polyclinic visits per person decreased 6- to 7-fold during this period.

**Conclusions:**

The significant reduction of the infectious disease burden; the doubling of the population size and the growth of the proportion of elderly individuals due to the declining death rates; the declining infant mortality rates to levels comparable to the national average as well as the decline in average numbers of polyclinic consultations per person, indicate that Medical Missions health service provision achieved its goal of improving the health and survival of the indigenous people by providing free, accessible and permanent medical services. Building upon this successful experience Medical Mission could be instrumental in addressing potential contemporary life-style related health threats.

The United Nations estimates that there are approximately 370 million indigenous peoples worldwide who live in at least seventy countries [1]. Many of them share an important unfortunate commonality, ie, persisting inequities in health status when compared to non-indigenous populations. As a result, total mortality as well as maternal and infant mortality, malnutrition, infectious diseases such as malaria and tuberculosis, non communicable diseases such as cardiovascular illnesses, HIV/AIDS, accidents and trauma, alcoholism, and suicidal behavior are disproportionally high in indigenous people [1,2]. These outcomes are for a large part attributable to poor living conditions in reservations and urban slums to which many of them have been assigned; unemployment; geographical isolation and financial barriers regarding access to mainstream health care, existing health prevention and promotion programs; and health services that are often insufficiently tuned to the cultural perceptions of the native people [2-4]. These conditions probably also apply to many of the approximately 43 million indigenous peoples of Latin America [2,3].

The current study focuses on the Amazonian Amerindians of the Republic of Suriname. This country is located on the northeast coast of South America and is part of the Amazon River Basin **(****Figure 1**). Its remote south and southwestern hinterlands are inhabited by Amerindian populations belonging predominantly to the Trio, Wayana, and Akurio tribes. This population, the majority of whom belong to the Trio tribe, numbered approximately 2500 persons in 2014 [5-7].

**Figure 1:**
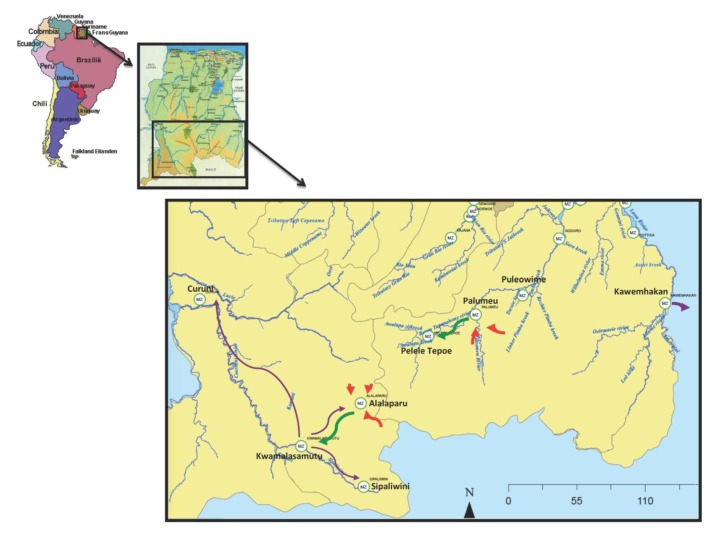
Positioning of Suriname in South America, and migrations of Amerindians in the south and southwestern parts of Suriname. (red – early 1960s; green – late 1960s-1970s; purple – 2005-2015).

The Amerindians traditionally lived in small communities of fifteen to fifty individuals who have familial ties. They moved regularly to new locations because of exhaustion of soil and game, ant plagues, family feuds, and deaths [5,6,8]. In contrast to many other Amerindian communities in the Western hemisphere, contacts with the “outside world” were initially rare. This situation changed with the advent of the missionaries in the 1950s followed by explorers, traders, and medical expeditions. The resulting deadly outbreaks of “cough diseases” and other infectious ailments due to exposure to infectious agents to which these populations had no immunity, led to the suspension of the medical expeditions and caused the Amerindians to move to the extreme south of Suriname where they maintained their traditional forager-horticulturalist way of living in an effort to avoid contact with the outside world [5,6,8].

Contacts with the “outside world” continued to occur sporadically until the 1960s, when “Operation Grasshopper” was launched [6,8]. This project was aimed at improving the infrastructure of Suriname by establishing airstrips in several villages in the interior. This led to the migration of the Amerindians to new locations and the formation of much larger villages such as Alalaparu and Palumeu established in 1963, and Tepu and Kwamalasamutu established around 1964 and 1978, respectively (**Figure 1**). However, an important disadvantage of living in larger groups was a more rapid spread of new pathogens and larger outbreaks of infectious diseases such as flu-like illnesses, tuberculosis, chicken pox, measles, and mumps during the late 1960s and the early 1970s [8,9].

The urgent need for more comprehensive health care was met by Medical Missions establishment of a structured health care system comprising a network of primary health care centers in the hinterland - including the southern and southwestern parts of Suriname - with referral if necessary to hospitals in Suriname’s capital city Paramaribo in the coastal area of the country [8,9]. This unique system was placed under the management of the non-governmental primary health care organization Medical Mission which is fully subsidized by the Surinamese government since 1972 [8,9].

Medical Mission has been providing these forest peoples free access to health care and culturally appropriate disease prevention and health promotion programs for the past fifty years. The polyclinics are all situated in the villages, and from the start health assistants were recruited from the local communities in order to avoid potential cultural misperceptions. Since the early 1980s, the health assistants receive a formal three-year training program that includes a wide range of basic patient care services including the dispensary of essential drugs, diagnostic tests, vaccinations, emergency and trauma care, mother and child care, including family planning services, as well as deliveries. Physicians and nurses of Medical Mission, whose headquarters are in the capital city Paramaribo, conduct regular supervisory visits to the clinics and support the health assistants who live nearby the clinics and run the daily services by short-wave radio or mobile telephone.

This paper provides an evaluation of the impact the unique health program of Medical Mission had on the health status of the Amazonian Amerindians. During the five decades of their resettlement into larger communities, these Amerindian peoples largely maintained their traditional life-style of hunting, fishing, and crop cultivation. These communities rely mainly on rain, creek or river water; electricity is generally not present in the villages aside from solar panels in the clinics and in some of the government facilities. Since the past decade, their increased involvement in trade and gold mining is affecting their way of living [10-12]. For this reason, the contemporary health challenges and those that lay ahead are also discussed.

## METHODS

### Study design and study population

This was a retrospective and descriptive study to evaluate Medical Mission’s primary health care program for its effectiveness on the health status of the indigenous Amerindian populations living in the southern and southwestern parts of Suriname. The study covered the period between 1965 and 2014. Temporal trends in infectious disease burden, population composition, death rates, including infant mortality rates, birth rates, as well as numbers of polyclinic consultations per person were used as proxies to assess the accomplishments of the program.

### Sources of data

From the start of Medical Mission in 1965, the physicians, nurses, and health assistants working with the indigenous communities have been responsible for the registration of all the villagers (name, gender, date of birth or estimated age), and for keeping track of numbers and purposes of patient visits to polyclinics, relocations as well as deaths and births. The health assistants have been instructed to tally all the polyclinic visit and also to register the first visit of an infectious disease episode of a patient. This information is recorded in weekly and monthly reports and submitted to Medical Mission headquarters in Paramaribo, where it is processed into year reports. Since the late 1990s, Medical Mission headquarters has automated its patient population database and weekly surveillance and monthly reports, which nowadays has replaced the previous paperwork and improved efficiency and speediness of data processing. Medical Mission headquarters, in its turn, submits death and birth certificates and surveillance reports to the relevant governmental health agencies. There is no official civilian registry in most of Suriname’s interior; therefore, Medical Mission’s records represent the only and most reliable sources of population data in Suriname’s hinterland.

Annual numbers of consultations due to diarrheal disease, respiratory tract infections, and malaria per year were retrieved from the annual reports for the periods 1965-1970, 1973-1977, 1982-1985 and from Medical Mission’s electronic weekly surveillance and monthly reports for the period 1997-2014. Annual numbers of births and deaths per year were retrieved from the year reports of Medical Mission for the periods 1965-1970, 1973-1977, 1982-1985, 1997-1999, as well as 2003-2010. Due to an internal civil war that lasted from 1986-1992 and ravaged the interior, including the health infrastructure, no data were available for the period 1986-1997. It took Medical Mission approximately 5 post-war years to rebuild its operations. Infant mortality rates were obtained from the available annual reports of 1965-1970 and 1982-1984 and for the period 2007-2014 by reviewing the available death certificates per year of infants (0-11 months) which are kept at Medical Missions headquarters.

Data on population composition by gender and age group were obtained from the annual report of 1969 and from the electronic population database of 2014. Estimated end-of-the year population sizes were collected from Medical Mission’s annual reports over the above-mentioned periods and from the electronic files. Total numbers of patient consultations per year were collected from the annual reports and the electronic monthly reports.

### Data processing

Yearly incidence rates for respiratory tract infections, diarrheal disease, and malaria were derived by dividing the number of cases in a particular year by the size of the estimated end-of-the year population in that year, and expressed per 1000 population per year. Averages covering three year intervals were compared to smooth for yearly fluctuations due to the relatively small population size. The same process was used for the birth and death rates estimations comparing several time periods.

The total population growth as well as the growth per age group and gender, were estimated by calculating the population growth of the total population between 1969 and 2014 as a percentage of the population of 1969. Graphical displays of population by size, age group and gender for these years were produced and compared with each other.

Crude death and birth rates were calculated by dividing the number of deaths or births, respectively, in a particular year by the estimated size of the end-of-the year population in that year, and were expressed per 1000 population per year. Averages of crude mortality covering three-year periods were computed and compared as well. In order to compare the crude death rates of 1969 with 2014 (the only years for which populations compositions by age and sex were available), a standardized mortality ratio was calculated to allow for age-standardization.

Infant mortality rates were calculated by dividing the number of infant (0-11months) deaths by the total number of live births in a particular year and were expressed per 1000 live births. Additional to the birth rates, fertility rates were calculated for 1969 and 2014, by dividing the total yearly number of births by the total number of women in the child bearing age (15-44 years).The average number of polyclinic visits per person per year was estimated by dividing the number of visits in a particular year by the estimated size of the end-of-the year population in that year.

In all cases, the raw data were processed using Tableau Personal edition desktop 2016 version 9.2 (Tableau Software, Seattle, WA, USA) and SPSS V.21.0 (IBM, Armonk, NY, USA).

### Ethical considerations

The Ministry of Health (VG 004-14) and the Director of the Medical Mission (C050/14/EvE) approved the use of the medical records, polyclinic registries, automated databases, and year reports of the Medical Mission for the current study.

## RESULTS

### Temporal trends in population size and composition

**Figure 2:**
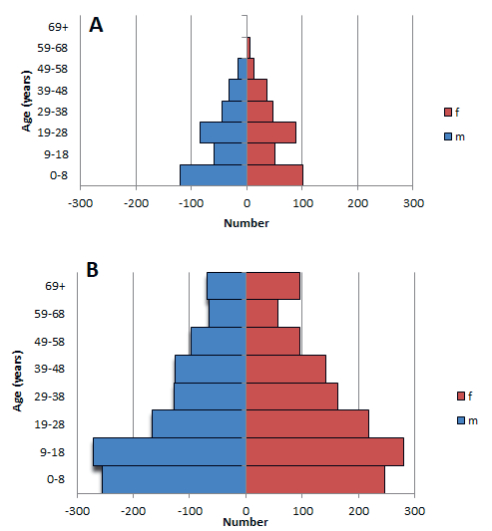
Population pyramids of the indigenous Amerindians from the southern and southwestern parts of Suriname, 1969 (**A**) and 2014 (**B**). f – female, m – male.

**Table 1** gives the numbers of males and females in several age groups of the southern and southwestern Amerindian populations in 1969 and 2014. When compared to 1969, the southern and southwestern Amerindian populations had more than doubled in size by 2014 (**Table 1**). Although growth had occurred in all age groups (**Table 1**), the average yearly growth in the group of 59 years and older was particularly evident. The observations not only suggest that these populations are increasing in size, but also that their age composition is changing so as to comprise proportionally more elderly individuals. This ageing of the population is illustrated by a comparison between the population pyramids of 1969 and 2014 (**Figure 2**), which shows not only better child survival but also better survival into old age.

**Table 1:** Average yearly incidence rates for respiratory tract infections, gastroenteritis, and malaria in indigenous Amerindians from the southern and southwestern of Suriname in 1969 and 2014*

Period	Respiratory tract infection	Gastroenteritis	Malaria
1968-1970	3123.6	885.0	53.8
1982-1985	200.1	103.2	211.7
1997-1999	659.5	374.1	240.3
2000-2002	644.4	438.5	396.2
2003-2005	793.5	534.1	517.7
2006-2008	983.5	458.1	40.3
2009-2011	781.7	328.4	21.5
2012-2014	739.8	383.0	1.9

*Data are per 1000 population per year.

### Temporal trends in incidence rates of infectious diseases

**Table 2** gives the yearly incidence rates of the main infectious diseases during various periods between 1968 and 2014 in the Amerindians from south and southwest Suriname. Respiratory tract infections were far more prevalent than gastroenteritis and malaria in 1968-1970 with more than three episodes per person per year. Their incidence had substantially declined over time (by about 75% by 2012-2014). Gastroenteritis occurred at a rate of about one-third of that of respiratory tract infections and had declined by about 53% by 2012-2014. As a result, both gastroenteritis and respiratory tract infections occurred in 2012-2014 at rates of less than one per person per year.

**Table 2:** Numbers of and average yearly growth by age group and gender in indigenous Amerindians from the southern and southwestern of Suriname in 1969 and 2014

	Males			Females		
**Age group (years)**	**Number in 1969**	**Number in 2014**	**Average yearly growth (%)**	**Number in 1969**	**Number in 2014**	**Average yearly growth (%)**
0-8	180	301	1.5	177	284	1.3
9-18	116	262	2.8	87	282	5.0
19-28	121	158	0.7	121	215	1.7
29-38	75	129	1.6	88	162	1.9
39-48	57	125	2.7	59	40	3.1
49-58	33	97	4.3	38	99	3.6
59+	16	136	16.7	15	153	20.4
Total	598	1208	2.3	585	1335	2.8

On the other hand, the average yearly incidence of malaria was relatively low in the period 1968-1970. However, it increased in the subsequent time periods to epidemic levels of almost 10 times higher than the initial value from the 1960s, then virtually vanished in the following decade (**Table 2**).

Overall, these results are consistent with a substantial decrease in the burden of the main infectious diseases in the indigenous populations in the south and southwest of Suriname during the fifty-year period covered by this study.

### Temporal trends in death and birth rates

**Table 3** depicts the trends in (crude) death rates, infant mortality, and birth rates in the southern and southwestern Amerindian populations over the period from 1965 through 2014. Average yearly crude death rates were roughly 12 to 16 per 1000 population per year in the 1960s, but had since then declined to on average 5 per 1000 population per year, ie, by approximately two-thirds. In order to standardize for the difference in age structure, the indirect standardization method was used to compare the death rate of 1969 with that of 2014. The crude death rates were 13.5 and 9.0 respectively and using the 2014 age-specific mortality rates as a reference, the standardized mortality ratio comparing 1969 with 2014 was 2.8.

**Table 3:** Yearly average death, birth and infant mortality rates, in indigenous Amerindians from the southern and southwestern of Suriname between 1965 and 2014*

Period	Average crude death rate	Average crude birth rate	Average infant mortality rate
1965-1967	15.9	39.8	63.7
1968-1970	12.2	43.8	32.4
1973-1977	4.9	32.8	N/A
1982-1985	6.3	25.7	15.5
1997-1999	4.4	22.1	N/A
2000-2002	6.2	28.9	N/A
2003-2005	5.3	29.7	N/A
2006-2008	5.9	32.0	20.4
2009-2011	2.8	30.2	8.4
2012-2014	5.0	26.5	28.9

N/A – not available

*Crude death rates are per 1000 population per year, infant mortality and crude birth rates are per 1000 live births per year.

The average infant mortality was almost 64 per 1000 live births per year in 1965-1967 (**Table 3**). Although there were wide fluctuations during the subsequent years, this value consistently remained under 50% of the initial rate (**Table 3**).

Average crude birth rates in the 1960s were roughly 40 live births per 1000 population per year (**Table 3**). This value was roughly 25% lower from the early 1980s on. The fertility rates for 1969 and 2014 were 140.5 and 91.1 per 1000 respectively.

Together, these results show that death rates, infant mortality rates, as well as birth rates had decreased in the past fifty years in the Amerindians populations, but that the decrease in death rates was much more pronounced than that in birth rates. These findings are consistent with the above-mentioned growth and shift in age composition of these communities over the period covered by this study.

### Temporal trends in average yearly polyclinic consultations per person

As shown in **Table 4**, the average number of yearly polyclinic consultations per person was about 28 in 1968-1970 (ie, almost three visits per month), approximately 10 in 1983-2003 (ie, about 1 visit per month), but about 3 in 2012-2014 (ie, roughly 1 visit every 4 months). These results clearly indicate a decline in the utilization of medical care despite the marked aging of the population.

**Table 4:** Average number of polyclinic visits per person per year in indigenous Amerindians from the southern and southwestern of Suriname between 1968 and 2014

Period	Average number of polyclinic visits per person per year
1968-1970	28.4
1973-1977	14.7
1982-1985	13.0
1997-1999	12.3
2000-2002	9.8
2003-2005	8.1
2006-2008	5.6
2009-2011	4.1
2012-2014	3.4

## DISCUSSION

This paper evaluates the impact of Medical Mission’s fifty-year old unique local health care provision on the health status of the indigenous Amerindian populations living in the southern and southwestern parts of Suriname. The results show a meaningful reduction of the infectious disease burden; substantially decreasing death rates, including infant mortality rates, and slower decreasing birth rates; a doubling of the population size and a growing proportion of elderly individuals; as well as a decline in average numbers of polyclinic consultations per person. Together, these observations indicate that the Medical Mission, as the sole provider of primary health care services, achieved its goal of providing free, accessible and effective medical services to the indigenous people from Suriname while taking into consideration their specific cultural perceptions.

The notable decrease in the burden of infectious diseases is indicated by the marked reduction in the incidence rates of the main infectious diseases, respiratory tract infections, gastroenteritis, and malaria. The relatively high incidence of respiratory tract infections in the late 1960s is consistent with reports mentioning that this group of diseases -recurrent epidemics of flu, outbreaks of measles, as well as surges in pneumonia and tuberculosis - represented major causes of illness and even death during the initial period of contact with outsiders, as mentioned earlier [8,9]. The substantial decrease in the occurrence of respiratory tract infections and gastroenteritis by the early 1980s was probably attributable to the availability of antibiotics treatment, the emergence of immunity, and, in the case of tuberculosis, isolation of patients in sanatoria in Paramaribo as well as mass screening, treatment, and BCG (Bacillus Calmette–Guérin) vaccination in the affected villages [8,9]. The decrease in the incidence of diarrheal disease is probably also attributable to the health promotional activities provided by the Medical Mission’s program. The fact that this decline was less pronounced when compared to that of respiratory tract infections may be explained by the greater risk of gastrointestinal infections in larger communities lacking safe piped water and adequate sanitary facilities, which is, unfortunately, still the case in most of Suriname’s hinterland [7]. The unexpected low reported values of gastroenteritis and respiratory tract infections in the early nineteen eighties compared to subsequent reporting periods could be due to the quality of reporting.

Malaria did not constitute a major disease burden in the indigenous populations in the 1960s. In fact, this disease was virtually under control in Suriname in those years [13]. However, from the late 1980s, malaria invaded the entire hinterland of the country including the southern and southwestern parts, causing massive epidemics in the Amerindian populations [14]. One of the important causes was the interior civil war between 1986 and 1992 which disrupted a large part of the primary health care infrastructure of Medical Mission [13]. The decline of malaria to near elimination levels during the first decade of the 21st century is therefore the more remarkable, and must be credited to increased funding to fight the disease, among others, by the Global Fund; the implementation of effective preventive measures including the use of impregnated bed nets; and the introduction of efficacious therapeutic regimens such as artemisinin-based combination therapy [14].

The shift in the composition of the Amerindian populations from small tribes characterized by a relatively small group of elderly individuals, a high birth rate, and a high death rate, towards larger population, lower death rates and a growing cohort of elderly, can probably for an important part attributed to the above-mentioned achievements in health care. The relatively low (crude) death rates for the reporting period between 1965 and 1970 is in accordance with the contention that the continuous availability medical care during the first five years following contact with outsiders averts the occurrence of massive numbers of contact-related deaths [15].

The advancements in maternal care, child vaccinations, and treatment of infectious diseases almost certainly have contributed to the meaningful reduction in infant mortality in the Surinamese Amerindians to around 20 per 1000 live births per year. This is comparable to the national infant mortality rate in Suriname [16] and is worth mentioning, particularly when considering that current rates of infant mortality in various Latin American countries exceed national averages by 3 to 4 times. [2,3,17-19]. Moreover, the rapid increase in the number of elderly people signifies the need for services that address the specific health care and social needs of this group [20,21].

The relatively high number of polyclinic visits between 1968 and 1970 (almost three per month per person) is probably attributable to the implementation in that period of the directly observed treatment short-course (so-called DOTS) strategy. This strategy was originally recommended by World Health Organization in order to maximize adherence to treatment for tuberculosis, and involved administering patients their daily medications under direct supervision of a health worker [22]. The use of this approach since the 1960s by Medical Mission for treating all infectious diseases in the indigenous communities explains the relatively high number of tallied polyclinic visits per person during particularly the first decades when regular outbreaks of these ailments occurred [8,9]. In general, the Amerindians were “polyclinic-minded” and made ample use of the services offered by Medical Mission [9]. As the DOTS strategy is still being applied for treating infectious diseases, the further reduction of patient visits to about one every three months in the period 2012-2014 is remarkable and clearly indicates a reduced need for medical treatment, which is consistent with the decrease in the occurrence of these conditions.

Obviously the current study is based entirely on Medical Mission’s data sources. It is possible that not all deaths have been reported to the polyclinics. However, since the communities where the polyclinics are situated are rather small, it is very unlikely that any death would remain unnoticed. To this date Medical Mission functions as registrar for the National Civilian Registry, for births and deaths in these remote communities. Self-management for infectious diseases could have increased over time in these communities, leading to a lower demand, but in our view not on such a scale to result in the sustained decline of the infectious disease burden over time. The demographic shift could have contributed to the further decline in the infectious disease burden, especially with respect to diarrhoeal and respiratory diseases. On the other hand malaria affects all ages, and the observed decline must thus be attributed to the anti-malaria activities carried out by Medical Mission.

### Limitations

Our data set is limited by the gaps between the reporting periods for which no annual reports were available either due to unclear reasons or the interruption of regular services by Medical Mission during the period of the internal civil war from 1986-1992. The automation of the reporting which started in 1997 could certainly have led to a more complete data set compared to the previous reporting periods. Moreover, training of staff and improvement of diagnostic capacity could have improved reporting as well. Despite all these factors however, the downward trends over time for most of the measured outcomes is significant.

Summarizing, the results from this study indicate that Medical Mission’s local primary health care structure has led to a declining burden of infectious diseases, declining birth, death rates and infant mortality rates in, as well as to a marked growth of the Amerindian populations in Suriname over the past fifty years. These developments have led to an accelerated epidemiological and a demographic transition which has also been observed in many other indigenous populations[23-25] and may fit Omram’s epidemiological transition from a stage of pestilences and epidemics (stage 1) to a stage of receding infectious disease burden (stage 2) facilitated by an increased availability of medical care [26-28]. In the meantime, the relatively modest consumption of medical care mentioned above might imply a gap in care for latent non-communicable diseases such as pre-diabetes, pre-hypertension, and premalignant conditions in these populations. If true, this signifies a need for screening and early intervention for these conditions. It is well documented that many indigenous peoples rapidly transitioned from high mortality from infectious diseases to high rates of premature mortality from non-communicable diseases, the so-called diseases of acculturation [2,17,20,29-33]. Indeed, our own preliminary studies (not shown) suggest that the proportion of deaths due to cardiovascular diseases and diabetes mellitus in Surinamese Amerindians have increased over the past decades. These observations suggest that these populations are at risk of transitioning to stage 3 as described by Omram [26], where infectious diseases would be replaced by chronic degenerative diseases. Whether this indeed holds true for the specific case of the Surinamese Amerindians should be addressed in more comprehensive future studies.

Building upon its fifty-year experience in successfully tackling infectious diseases Medical Mission could be instrumental in addressing these potential contemporary health threats. The network of polyclinics scattered over the hinterland and staffed by local trained health assistants is an important part in this decentralized model of care. On the global level health care delivery facilities in remotely living indigenous populations tend to be either absent or experience problems related to the recruitment of health care providers willing to live in those communities, which hampers the access to essential health care [34,35].

The Amerindian populations in the south and southwest of Suriname still maintain a predominantly traditional hunter-gatherer life-style that may protect them from early-onset non-communicable diseases. Recent studies have shown that the prevalence of obesity, tobacco use and physical inactivity are still relatively low among these populations [36,37]. This should be studied further and is important for the development of prevention policies.

## CONCLUSIONS

The significant reduction of the infectious disease burden; the doubling of the population size and the growth of the proportion of elderly individuals due to declining infant mortality and overall death rates; as well as the decline in average numbers of polyclinic consultations per person indicate that Medical Mission’s program achieved its goal of impacting the health and survival of the indigenous people over the past fifty years by establishing a permanent accessible and free medical health care system. Building upon this experience in successfully tackling infectious, diseases, Medical Mission could be instrumental in addressing life style related potential contemporary health threats of these indigenous people.
